# Real-World Clinical Effectiveness and Safety of Vedolizumab and Ustekinumab in Biologic-Naïve Patients With Early or Late Crohn’s Disease: Results From the EVOLVE Expansion Study

**DOI:** 10.1093/crocol/otaf031

**Published:** 2025-07-09

**Authors:** Britt Christensen, Michael Scharl, Brian Bressler, Zaeem Khan, Yuliya Halchenko, Celine Gisler, Pravin Kamble, Shashi Adsul, Zeinab Farhat, Marc Ferrante

**Affiliations:** Department of Gastroenterology, The Royal Melbourne Hospital, Melbourne, VIC, Australia; Department of Medicine, The University of Melbourne, Melbourne, VIC, Australia; Department of Gastroenterology and Hepatology, University Hospital Zurich, University of Zurich, Zurich, Switzerland; Department of Medicine, Division of Gastroenterology, St. Paul’s Hospital, Vancouver, BC, Canada; Epidemiology and Scientific Affairs, PPD, Part of Thermo Fisher Scientific, Montreal, QC, Canada; Epidemiology and Scientific Affairs, PPD, Part of Thermo Fisher Scientific, Montreal, QC, Canada; Department of Gastroenterology and Hepatology, University Hospital Zurich, University of Zurich, Zurich, Switzerland; Global Evidence and Outcomes, Takeda, Cambridge, MA, United States; Global Evidence and Outcomes, Takeda, Cambridge, MA, United States; Global Evidence and Outcomes, Takeda, Cambridge, MA, United States; Department of Gastroenterology and Hepatology, University Hospitals Leuven, KU Leuven, Leuven, Belgium

**Keywords:** biologics, Crohn’s disease, disease duration, inflammatory bowel disease, effectiveness

## Abstract

**Background:**

We evaluated the real-world effectiveness and safety of vedolizumab and ustekinumab as first-line biologics in patients with Crohn’s disease (CD), by disease duration.

**Methods:**

EVOLVE Expansion (ClinicalTrials.gov, NCT05056441) was a retrospective medical chart review study in Australia, Belgium, and Switzerland. Biologic-naïve patients with CD (≥18 years old) initiated first-line biologic treatment with vedolizumab or ustekinumab. Cumulative rates of clinical response, remission, mucosal healing, and treatment persistence were assessed over 36 months. Outcomes were compared between patients with early (≤2 years) and late (>2 years) disease at biologic initiation. Serious adverse events (SAEs), serious infections (SIs), and healthcare resource use (HCRU) were evaluated.

**Results:**

In early (*n* = 249) and late (*n* = 371) CD subgroups, there were no significant differences over 36 months between vedolizumab and ustekinumab in the cumulative rates of clinical response (early CD, 81.6% vs 80.7%; *P* = .31; late CD, 83.7% vs 86.5%; *P* = .31) or clinical remission (early CD, 87.9% vs 85.0%; *P* = .74; late CD, 91.1% vs 90.6%; *P* = .96). In patients with early CD, mucosal healing rates were significantly higher with vedolizumab than ustekinumab at both 24 (*P* = .02) and 36 months (*P* = .03). Treatment persistence was significantly higher with ustekinumab versus vedolizumab over 36 months in patients with late CD, but similar in patients with early CD. There were no significant differences in SAEs, SIs, or HCRU.

**Conclusions:**

Both vedolizumab and ustekinumab are effective treatment options for early- and late-stage CD. Over 36 months, mucosal healing rates in patients with early CD were significantly higher with vedolizumab than with ustekinumab.

## Graphical Abstract

**Graphical Abstract:**
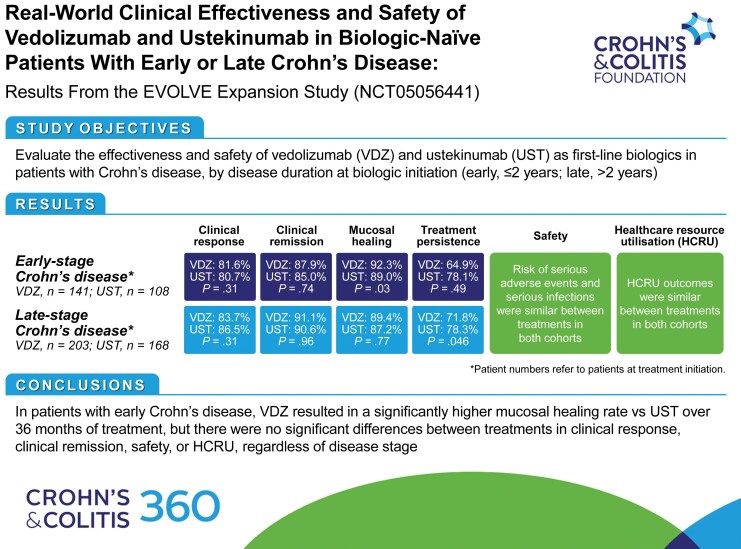

**Key Messages**

**What is already known?**

Vedolizumab and ustekinumab are novel biologics that are approved for the treatment of Crohn’s disease.

**What is new here?**

In patients with early (≤2 years’ duration) Crohn’s disease at biologic initiation, vedolizumab resulted in a significantly higher rate of mucosal healing versus ustekinumab over 36 months, but there were no significant differences between treatments in clinical response, clinical remission, safety, or healthcare resource use, regardless of disease stage.

**How can this study help patient care?**

Both vedolizumab and ustekinumab can be considered as treatment options for patients with early- and late-stage Crohn’s disease.

## Introduction

Crohn’s disease (CD) is a chronic relapsing-remitting inflammatory condition that affects the gastrointestinal tract and requires lifelong treatment.^1,2^ The persistence or recurrence of inflammation can result in progressive damage to the gastrointestinal tract, with approximately 40% of patients with CD requiring surgery within 10 years of diagnosis.^3–5^ Chronic inflammation in the affected gut can take time to develop into clinically significant manifestations. Therefore, patients may experience a lag time between the onset of inflammation and the onset of signs and symptoms.^6^ If clinically active inflammation is diagnosed before complications develop, there is a window of opportunity to treat the inflammation, during which the likelihood of treatment success is greatest.^7,8^ Early diagnosis and prompt initiation of treatment may prevent irreversible bowel damage and help preserve the quality of life.^6^

Several clinical studies have demonstrated the benefits of initiating biologic therapies within 2 years of diagnosis, including enhanced remission maintenance and decreased CD-related complications.^9,10^ A real-world study found that initiating antitumor necrosis factor (TNF) therapy within the first 2 years of diagnosis was associated with a low rate of CD-related complications (7.1% had progression in the Montreal classification of disease behavior), as well as high rates of clinical (85.1%) and endoscopic remission (49.5%). However, no differences were seen with different timing of interventions within this 2-year period and the study did not include patients who started therapy after 2 years of diagnosis.^11^ The early use of vedolizumab to reduce the risk of CD-related surgery is supported by a post hoc analysis of the GEMINI 2 and 3 studies (*n* = 1253), which explored the effect of vedolizumab treatment on the risk of CD-related surgery at ≤2 years versus >2 years or ≤5 years versus >5 years since diagnosis, after accounting for the probability of vedolizumab response based on previous bowel surgery, anti-TNF use, fistulizing disease, baseline albumin, and baseline C-reactive protein levels.^12^ Patients with a low or intermediate probability of vedolizumab response, as classified with a validated clinical decision support tool, had a greater risk of CD-related surgery than patients with a high probability of response.^12^ In this group, there was a trend of lower risk of surgery for those treated earlier compared with those treated later (≤2 years vs >2 years, odds ratio 0.77 [95% CI: 0.38-1.58]; ≤5 years vs >5 years, odds ratio 0.61 [95% CI: 0.37-1.00]).^12^ Taken together, these findings emphasize the critical window of opportunity within the initial years following diagnosis, highlighting the potential to optimize patient outcomes through timely therapeutic interventions. Further research is warranted to elucidate optimal treatment algorithms and long-term outcomes in CD management.

Several biologics have been approved for the treatment of patients with moderate to severe CD, including vedolizumab and ustekinumab. Vedolizumab is a gut-focused antilymphocyte trafficking α_4_β_7_-integrin inhibitor,^13,14^ which was approved for the treatment of moderate to severe CD based on the results of the phase 3 GEMINI 2 and GEMINI 3 trials in anti-TNF-naïve or anti-TNF-experienced patients.^15^ Ustekinumab is an interleukin-12/23 p40 inhibitor,^16^ which was approved based on the efficacy and safety results of its phase 3 clinical trials in patients with moderate to severe CD, with significantly higher rates of clinical response and clinical remission versus placebo.^16,17^

Further research is required to determine the comparative real-world effectiveness and safety of these agents and thus enable physicians to determine which agents are best suited for first-line biologic treatment for individual patients. The EVOLVE Expansion (ClinicalTrials.gov, NCT05056441)^18^ real-world study in biologic-naïve patients with CD reported similar rates of clinical response and clinical remission, and a similar risk of serious adverse events (SAEs) in patients who received first-line biologic treatment with vedolizumab or ustekinumab. More real-world data are needed with regard to the outcomes of biologic use in patients with early versus late CD. The aim of this subanalysis of the EVOLVE Expansion study^18^ was to evaluate the real-world clinical effectiveness and safety of vedolizumab and ustekinumab as first-line biologics in biologic-naïve patients, by disease duration.

## Methods

### Study Design

The design of the EVOLVE Expansion study has been described previously (Ferrante, submitted 2024).^18^ Briefly, this was a multicenter, observational, retrospective medical chart review of patients with CD who initiated first-line biologic treatment with vedolizumab or ustekinumab during the eligibility period (March 1, 2017, to May 2021 for Australia; November 11, 2016, to May 2021 for Belgium; and June 2, 2017, to May 2021 for Switzerland). The date of vedolizumab or ustekinumab initiation was defined as the index date. Because vedolizumab was approved earlier than ustekinumab in all 3 countries, the start dates of the eligibility period aligned with the market approval of ustekinumab by local regulatory authorities. Data were collected post-initiation of vedolizumab or ustekinumab until chart abstraction, treatment discontinuation, death, or loss to follow-up, whichever occurred first. Subgroups of patients were included in this analysis based on patients’ disease duration. Early CD was defined as a disease duration of ≤2 years between CD diagnosis and treatment with biological therapy, and late CD was defined as a disease duration of >2 years between CD diagnosis and treatment with biological therapy.

The study was conducted in accordance with the Declaration of Helsinki, Good Pharmacoepidemiology Practice, The International Society for Pharmacoepidemiology Good Pharmacoepidemiology Practice guidelines, and local regulations. This study was approved by institutional review boards/ethics committees of the participating institutions. All patients from Australia and Switzerland included in the study provided informed consent; Belgium has obtained a waiver of consent for retrospective studies, hence consent was not sought in this country.

### Study Population

Eligible participants were biologic-naïve patients aged ≥18 years with previously diagnosed CD who initiated treatment with vedolizumab or ustekinumab in Australia, Belgium, or Switzerland during the eligibility period. Patients had to complete the induction phase of treatment and have ≥6 months of follow-up data from the start of treatment (index date) to chart abstraction initiation. Exclusion criteria included previous biologic treatment for CD or any other conditions at any point during the patient’s lifetime, treatment with vedolizumab or ustekinumab as part of a clinical trial, initiation of combination therapy with 2 biologic agents, or induction treatment with a subcutaneous formulation of ustekinumab only (Supplementary Data, Figure S1).

Given the observational nature of this study, treatments were administered to patients at the discretion of the treating physician and according to the treatment label. Vedolizumab 300 mg was administered intravenously at weeks 0, 2, and 6, then every 8 weeks thereafter. For ustekinumab, a single dose was administered intravenously at week 0 based on participants’ body weight (260 mg for ≤55 kg body weight, 390 mg for >55 kg to ≤85 kg body weight, and 520 mg for >85 kg body weight); 8 weeks after the initial intravenous dose, ustekinumab 90 mg was administered subcutaneously, then every 8 weeks thereafter. During 36 months of follow-up in the EVOLVE Expansion study, the majority of patients (vedolizumab, 91.6%; ustekinumab, 91.3%) received maintenance dosing every 8 weeks; only a small percentage of patients received maintenance dosing every 12 weeks (vedolizumab, 0.9%; ustekinumab, 2.2%).^18^

### Study Assessments

Data were collected retrospectively from patients’ medical charts. Baseline data included patient demographics, prior non-biologic treatments up to 12 months before index, laboratory measures (most recent from 3 months prior to index), CD characteristics at diagnosis and index, disease activity, and exacerbations from 6 months prior to index. In addition, preexisting extraintestinal manifestations and comorbidities from 12 months prior to index, healthcare resource use (HCRU) prior to index, and concomitant non-biologic CD treatments were collected at baseline. Clinical outcomes, treatment patterns, safety, and HCRU were all collected after the index date.

### Study Outcomes

#### Effectiveness

Cumulative rates of clinical outcomes (clinical response, clinical remission, and mucosal healing) and treatment patterns (persistence) over 36 months were assessed using hierarchical algorithms and were estimated using a time-to-event analysis with the Kaplan–Meier method. To be included in an analysis for a specific effectiveness or treatment persistence outcome, patients had to have ≥1 record on that specific outcome; patients with no records on a specific outcome were excluded from the analysis of that outcome.

Clinical response was defined using the algorithm: (1) a positive change in CD activity index category (<150, 151-219, 220-450, or >450) from baseline OR if unknown; (2) a decrease of ≥3 points from baseline in the Harvey-Bradshaw Index (HBI) OR if unknown; (3) a decrease of ≥3 points from baseline in the modified HBI (excluding the HBI subscale for abdominal mass) OR if unknown; (4) treatment response recorded in the medical chart as “complete response” or “partial response.”

Clinical remission was defined using the algorithm: (1) CD activity index score of <150 points OR if unknown; (2) HBI score of ≤4 OR if unknown; (3) modified HBI score of ≤4 OR if unknown; (4) remission status recorded in the medical chart as “in remission.”

Mucosal healing was defined using the algorithm: (1) endoscopic assessment score of 0 or 1 (ie, normal or inactive disease or mild disease) OR if unknown; (2) Simple Endoscopic Score for CD of <3 OR if unknown; (3) “lack of ulceration” defined by ≥1 of the following endoscopic procedure finding(s) from the case report form drop-down list—either selection of “no ulcers” or free-text indication of “lack of ulceration” OR if unknown; (4) ≥1 non-endoscopic procedure finding(s) from the drop-down list indicating inactive disease (no findings/no active disease, no erosion, no ulcers, no inflammation or inflammatory activity, or no pathological findings).

Treatment patterns were assessed as treatment persistence, defined as the time from index treatment initiation to the end of the follow-up period or discontinuation of index treatment, whichever was earliest. Dose escalation was defined as an increase in dose frequency for ≥2 consecutive infusions/injections.

#### Safety/tolerability

Rates of SAEs and serious infections (SIs) were assessed within 5 half-lives post-treatment discontinuation (vedolizumab, 126 days; ustekinumab, 95 days). SAEs and SIs were classified as serious if they were life-threatening, required hospitalization, resulted in significant disability/incapacity, or were recorded as an important medical event.

#### Healthcare resource use

HCRU outcomes included rates of CD exacerbations, CD-related surgeries, and CD-related hospitalizations.

### Statistical Analyses

Data were compared between vedolizumab and ustekinumab treatment cohorts in 2 patient subgroups: those with early CD (≤2 years) and those with late CD (>2 years). Continuous data were described using mean, SD, median, and interquartile range; categorical variables were described as frequencies and percentages. Unadjusted comparisons were performed using *t*-tests or non-parametric tests for continuous data, and chi-square tests for categorical data. Cumulative rates during 36 months of treatment for effectiveness outcomes (clinical response, clinical remission, and mucosal healing) and the treatment persistence were calculated using Kaplan–Meier analyses; estimates at months 12, 24, and 36 were analyzed. Unadjusted *P* values were calculated using the log-rank test. The electronic data capture system did not permit questions to be unanswered, with data being reported as unknown if not available (including “missing,” “no data,” and “unspecified”) in the medical chart. Except for the imputation of some partial missing date values, no other imputation of missing values was undertaken.

Safety and HCRU outcomes were analyzed as incidence rates of first occurrences per 100 patient-years and hazard ratios (HRs) with 95% CI. All adjusted analyses were performed using inverse probability of treatment weighting (IPTW) methodology to account for differences in baseline characteristics between treatment groups. The baseline covariates included in the IPTW model were age at index treatment initiation, sex, disease location, disease duration, pre-index disease-related hospitalizations, disease severity at index, steroid dependency status at index, fistula status at index, biochemical index, disease behavior, year of index treatment initiation, and country. Disease severity (remission, mild disease, moderate disease, severe disease, or unknown) was classified using predefined algorithms based on disease activity measures: (1) CD activity index or if unknown, (2) HBI or if unknown, (3) modified HBI or if unknown, (4) physician global assessment score.

To determine the biochemical index as being within normal range or outside normal range, laboratory measures (closest within 3 months before the date of index treatment initiation) for C‑reactive protein, fecal calprotectin, and serum albumin were used. Each laboratory measure was classified as being within the normal range, outside of the normal range, or unknown, using local reference ranges from the study sites. Indicators of baseline biochemical disease were based on the available data using a hierarchical algorithm in the following descending order of preference: fecal calprotectin ≥250 mg/kg, followed by C-reactive protein ≥5 mg/L, followed by albumin <35 g/L. The first available indicator determined whether the patient was “within the normal range” or “outside of the normal range” according to this hierarchy.

A *P* value of <.05 was used to determine statistical significance. All data analyses were carried out using SAS version 9.4 or higher.

## Results

### Baseline Characteristics

A total of 249 patients had early CD (vedolizumab, *n* = 141; ustekinumab, *n* = 108) and 371 patients had late CD (vedolizumab, *n* = 203; ustekinumab, *n* = 168; Figure S1). There were no statistically significant differences between treatment groups for smoking status or BMI, with smoking status included in the IPTW model as a covariate. At baseline in early CD, 18 of 141 vedolizumab-treated patients underwent a total of 24 CD-related surgeries and 9 of 108 ustekinumab-treated patients had 9 CD-related surgeries (Table 1). At baseline in late CD, 52 of 203 vedolizumab-treated patients underwent 82 CD-related surgeries and 51 of 168 ustekinumab-treated patients had 87 CD-related surgeries (Table 2). The most common types of pre-index CD-related surgeries in early CD were ileocolonic bowel resection (vedolizumab, 66.7%; ustekinumab, 55.6%) and ileostomy reversal (vedolizumab, 16.7%; ustekinumab, 0%; Table 1). A greater range of pre-index CD-related surgeries occurred in late CD, including ileocolonic bowel resection (vedolizumab, 48.8%; ustekinumab, 50.6%), segmental small bowel resection (vedolizumab, 14.6%; ustekinumab, 13.8%) and other surgeries (vedolizumab, 14.6%; ustekinumab, 16.1%; Table 2). After IPTW, the treatment groups were well-balanced in both the early CD (Table 1) and late CD cohorts (Table 2).

**Table 1.** Baseline demographics and disease characteristics in patients with early CD (≤2 years), by treatment.

Characteristic	Unweighted			IPTW weighted		
	Vedolizumab (*n* = 141)	Ustekinumab (*n* = 108)	*P* value	Vedolizumab (*n* = 125)	Ustekinumab (*n* = 124)	Standardized difference after IPTW
Age, mean ± SD, y	44.5 ± 19.1	39.9 ± 18.5	.0567	42.8 ± 18.5	43.1 ± 18.9	−0.0143
Sex, male, *n* (%)	72 (51.1)	54 (50.0)	.8678	66 (52.5)	62 (49.5)	−0.0591
BMI, *n* (%)	136 (96.5)	104 (96.2)	.3229	122 (97.6)	123 (96.0)	
<18.5	5 (3.7)	4 (3.8)		4 (3.2)	3 (2.4)	
18.5–25.0	64 (47.1)	58 (55.8)		60 (49.5)	65 (53.7)	
25.0–29.0	46 (33.8)	25 (24.0)		39 (31.5)	29 (24.6)	
≥30	21 (15.4)	17 (16.3)		19 (15.3)	22 (18.3)	
Smoking status, *n* (%)	141 (100)	108 (100)	.9772	125 (100)	124 (100)	
Current	29 (20.6)	22 (20.4)		24 (19.4)	29 (23.6)	−0.1011
Former	25 (17.7)	20 (18.5)		19 (14.9)	17 (13.5)	0.0381
Never smoked	73 (51.8)	57 (52.8)		66 (55.2)	66 (53.3)	−0.0135
Unknown	14 (9.9)	9 (8.3)		16 (13.1)	12 (9.46)	
Disease duration, median (min, max), y	0.5 (0.0, 2.0)	0.5 (0.0, 1.9)	.7667	0.5 (0.0, 2.0)	0.5 (0.0, 1.9)	0.0632
Follow-up period, median (min, max), mo	29.1 (5.9, 77.1)	23.4 (3.3, 57.5)	.008	28.0 (5.9, 77.1)	28.4 (3.3, 57.5)	0.0084
CD location^a^ at index, *n* (%)			.0152			
L1 (+/– L4)	71 (50.4)	60 (55.6)		68 (54.6)	72 (58.1)	−0.0701
L2 (+/– L4)	28 (19.9)	11 (10.2)		18 (14.4)	14 (11.3)	0.0927
L3 (+/– L4)	34 (24.1)	37 (34.3)		32 (25.9)	38 (30.6)	−0.1045
Disease behavior^a^ at index,^b^*n* (%)			.3313			0.1024
B1 (+/– p)	104 (73.8)	70 (64.8)		92 (73.4)	86 (69.4)	0.0880
B2 (+/– p)	19 (13.5)	24 (22.2)		19 (15.4)	21 (17.2)	−0.0483
B3 (+/– p)	10 (7.1)	8 (7.4)		7 (5.9)	8 (6.4)	−0.0235
Active fistula^c^ at index, *n* (%)	11 (7.8)	5 (4.6)	.3118	8 (6.1)	7 (6.0)	−0.0445
Disease severity at index,^b^*n* (%)			.0631			
Normal	10 (7.1)	10 (9.3)		9 (7.3)	15 (11.8)	
Mild	40 (28.4)	14 (13.0)		38 (30.7)	17 (13.6)	−0.1781
Moderate	64 (45.4)	62 (57.4)		55 (43.7)	66 (52.8)	0.1292
Severe	17 (12.1)	13 (12.0)		15 (12.4)	15 (11.9)	−0.0989
Prior non-biologic therapy, *n* (%)	123 (87.2)	97 (89.8)	.5292	111 (88.5)	109 (88.0)	0.0170
Steroid dependent, *n* (%)	28 (19.9)	15 (13.9)	.0047	20 (16.2)	20 (16.3)	0.0038
Biochemical index,^d^*n* (%)	141 (100)	108 (100)	.0084			
Within normal range	44 (31.2)	18 (16.7)		29 (23.1)	25 (20.3)	0.0691
Outside normal range	64 (45.4)	69 (63.9)		67 (53.4)	69 (55.5)	−0.0425
Unknown	33 (23.4)	21 (19.4)		29 (23.5)	30 (24.2)	
Prior CD-related surgeries (from CD diagnosis), *n* (%)	18 (12.8)	9 (8.3)	.2649	13 (10.5)	12 (9.9)	0.0206
Type of CD-related surgeries*, n* (%)						
*n*	24	9				
Ileocolonic bowel resection	16 (66.7)	5 (55.6)				
Segmental small bowel resection	0	1 (11.1)				
Small bowel resection with stoma (temporary)	1 (4.2)	0				
Small bowel resection with stoma (permanent)	0	0				
Stricturoplasty	1 (4.2)	0				
Perianal surgery/fistula-related	1 (4.2)	1 (11.1)				
Ileostomy reversal	4 (16.7)	0				
Other	1 (4.2)	2 (22.2)				
CD-related hospitalizations (12 months prior), *n* (%)	29 (20.6)	24 (22.2)	.7519	27 (21.6)	24 (19.0)	0.0643

*P* < .05 was considered a statistically significant difference.

^a^Phenotype is according to the Montreal classification. Disease location was defined as ileal involvement (L1), colonic involvement (L2), ileocolonic involvement (L3), or upper gastrointestinal disease (L4). Disease behavior was defined as non-stricturing or non-penetrating (B1), stricturing (B2), or penetrating (B3) or perianal disease (p).

^b^Numbers with unknown status are not shown.

^c^Includes enterocutaneous, perianal, rectovaginal, other, or unknown.

^d^To determine whether the biochemical index was within normal range or outside normal range, laboratory measures (closest within 3 months before the date of index treatment initiation) for C‑reactive protein, fecal calprotectin, and serum albumin were used. Each laboratory measure was classified as being within normal range, outside of normal range, or unknown, using local reference ranges from the study sites. A hierarchical algorithm that gave preference to the evaluation of (1) fecal calprotectin, (2) C-reactive protein, and (3) serum albumin to determine the biochemical composite marker was then applied. If all 3 laboratory measures were available for an individual patient, biochemical composite marker evaluation was determined by that patient’s fecal calprotectin.

Abbreviations: CD, Crohn’s disease; IPTW, inverse probability treatment weighting.

**Table 2.** Baseline demographics and disease characteristics in patients with late (>2 years) CD, by treatment.

Characteristic	Unweighted			IPTW weighted		
	Vedolizumab (*n *= 203)	Ustekinumab (*n* = 168)	*P* value	Vedolizumab (*n* = 184)	Ustekinumab (*n* = 187)	Standardized difference after IPTW
Age, mean ± SD, y	50.3 ± 16.2	47.9 ± 15.5	.1574	49.4 ± 15.8	50.3 ± 16.1	0.0602
Sex, male, *n* (%)	100 (49.3)	90 (53.6)	.4084	93 (50.6)	92 (49.2)	−0.0265
BMI, *n* (%)	196 (96.6)	166 (98.8)	.7723	175 (95.1)	185 (98.9)	
<18.5	10 (5.1)	3 (1.8)		8 (4.7)	3 (1.8)	
18.5-25.0	89 (45.4)	84 (50.6)		78 (44.4)	92 (49.6)	
25.0-29.0	65 (33.2)	47 (28.3)		61 (34.8)	56 (30.4)	
≥30	32 (16.3)	32 (19.3)		28 (16.1)	34 (18.3)	
Smoking status, *n* (%)	203 (100)	168 (100)				
Current	38 (18.7)	24 (14.3)	.2059	30 (16.5)	24 (12.7)	0.1089
Former	48 (23.6)	32 (19.0)		46 (25.0)	40 (21.5)	0.0831
Never smoked	98 (48.3)	87 (51.8)		90 (49.1)	97 (51.9)	−0.0566
Unknown	19 (9.4)	25 (14.9)		17 (9.4)	26 (13.9)	
Disease duration, median (min, max), y	10.4 (2.0, 53.1)	12.1 (2.1, 46.0)	.3436	10.5 (2.0, 53.1)	11.0 (2.1, 46.0)	0.0399
Follow-up period, median (min, max), mo	32.2 (4.5, 79.4)	23.0 (2.8, 62.0)	<.0001	26.6 (4.5, 79.4)	26.5 (2.8, 62.0)	0.0577
CD location^a^ at index, *n* (%)			.0179			
L1 (+/– L4)	87 (42.9)	75 (44.6)		80 (43.2)	76 (40.9)	0.0475
L2 (+/– L4)	53 (26.1)	26 (15.5)		42 (22.5)	43 (22.9)	−0.0086
L3 (+/– L4)	57 (28.1)	57 (33.9)		57 (41.0)	60 (32.2)	−0.0255
Disease behavior^a^ at index,^b^*n* (%)			.0625			
B1 (+/– p)	124 (61.1)	86 (51.2)		107 (57.8)	100 (53.4)	0.0891
B2 (+/– p)	61 (30.0)	58 (34.5)		56 (30.3)	62 (33.0)	−0.0587
B3 (+/– p)	9 (4.4)	18 (10.7)		14 (7.6)	14 (7.4)	0.0086
Active fistula^c^ at index, *n* (%)	24 (11.8)	29 (17.3)	.1361	22 (12.1)	27 (14.3)	−0.0107
Disease severity at index,^b^*n* (%)			.5206			
Normal	24 (11.8)	13 (7.7)		21 (11.2)	16 (8.8)	
Mild	33 (16.3)	33 (19.6)		32 (17.2)	33 (17.6)	0.0150
Moderate	111 (54.7)	95 (56.5)		100 (54.4)	109 (58.3)	−0.0198
Severe	16 (7.9)	9 (5.4)		14 (7.7)	14 (7.7)	0.0055
Prior non-biologic therapy, *n* (%)	152 (74.9)	122 (72.6)	.6223	139 (75.2)	141 (75.5)	0.0068
Steroid dependent, *n* (%)	32 (15.8)	15 (8.9)	.0092	26 (14.0)	23 (12.1)	0.0554
Biochemical index,^d^*n (%)*	203 (100)	168 (100)	.6242	185 (100)	186 (100)	
Within normal range	72 (35.5)	57 (33.9)		66 (35.7)	70 (37.6)	−0.0393
Outside normal range	75 (46.9)	70 (41.7)		70 (37.8)	71 (38.0)	−0.0055
Unknown	56 (27.6)	41 (24.4)		49 (26.5)	46 (24.6)	
Prior CD-related surgeries (from CD diagnosis), *n* (%)	52 (25.6)	51 (30.4)	.3101	52 (28.3)	55 (29.5)	0.0271
Type of CD-related surgeries, *n* (%)						
*n*	82	87				
Ileocolonic bowel resection	40 (48.8)	44 (50.6)				
Segmental small bowel resection	12 (14.6)	12 (13.8)				
Small bowel resection with stoma (temporary)	5 (6.1)	2 (2.3)				
Small bowel resection with stoma (permanent)	0	1 (1.1)				
Stricturoplasty	3 (3.7)	5 (5.7)				
Perianal surgery/fistula-related	7 (8.5)	7 (8.0)				
Ileostomy reversal	3 (3.7)	2 (2.3)				
Other	12 (14.6)	14 (16.1)				
CD-related hospitalizations (12 months prior), *n* (%)	23 (11.3)	30 (17.9)	.0737	25 (13.5)	25 (13.2)	0.0065

*P* < .05 was considered a statistically significant difference.

^a^Phenotype is according to the Montreal classification. Disease location was defined as ileal involvement (L1), colonic involvement (L2), ileocolonic involvement (L3), or upper gastrointestinal disease (L4). Disease behavior was defined as non-stricturing or non-penetrating (B1), stricturing (B2), or penetrating (B3) or perianal disease (p).

^b^Numbers with unknown status are not shown.

^c^Includes enterocutaneous, perianal, rectovaginal, other, or unknown.

^d^To determine whether the biochemical index was within normal range or outside normal range, laboratory measures (closest within 3 months before the date of index treatment initiation) for C‑reactive protein, fecal calprotectin, and serum albumin were used. Each laboratory measure was classified as being within normal range, outside of normal range, or unknown, using local reference ranges from the study sites. A hierarchical algorithm that gave preference to the evaluation of (1) fecal calprotectin, (2) C-reactive protein, and (3) serum albumin to determine the biochemical composite marker was then applied. If all 3 laboratory measures were available for an individual patient, biochemical composite marker evaluation was determined by that patient’s fecal calprotection.

Abbreviations: CD, Crohn’s disease; IPTW, inverse probability treatment weighting.

### Concomitant Therapies During the Study

A number of patients were dependent on corticosteroids at index treatment initiation (early CD: vedolizumab, 19.9%; ustekinumab, 13.9%; late CD: vedolizumab, 15.8%; ustekinumab, 8.9%) (Tables 1 and 2). In the early CD subgroup, 14.9% of vedolizumab-treated patients and 14.8% of ustekinumab-treated patients augmented index treatment with corticosteroids, while 36.2% of vedolizumab-treated patients and 25.9% of ustekinumab-treated patients discontinued corticosteroid use (*P* = NS). In the late CD subgroup, 17.2% of vedolizumab-treated patients and 6.0% of ustekinumab-treated patients augmented index treatment with corticosteroids, while 24.1% of vedolizumab-treated patients and 11.9% of ustekinumab-treated patients discontinued corticosteroid use (*P* = NS).

In the early CD subgroup, 42.6% of vedolizumab-treated patients and 44.4% of ustekinumab-treated patients did not require non-biologic therapy, including steroids, during the post-index period (*P* = NS). In the late CD subgroup, 45.8% of vedolizumab-treated patients and 51.2% of ustekinumab-treated patients did not require non-biologic therapy during the post-index period (*P* = NS).

In the early CD subgroup, prior to initiating index treatment, 31.9% of vedolizumab-treated patients and 42.6% of ustekinumab-treated patients were using immunomodulators or immunosuppressives (*P* = .0253). Only 2.8% of patients in both treatment groups augmented index treatment with these non-biological therapies, while 6.4% of vedolizumab-treated patients and 11.1% of ustekinumab-treated patients discontinued their use (*P* = NS).

In the late CD subgroup, prior to initiating index treatment, 38.9% of patients on vedolizumab and 47.0% of patients on ustekinumab were using immunomodulators or immunosuppressives (*P* = NS). After initiating index treatment, 1.0% of vedolizumab-treated patients and 3.6% of ustekinumab-treated patients augmented treatment with these non-biological therapies, and 22.7% of vedolizumab-treated patients and 21.4% of ustekinumab-treated patients discontinued their use (*P* = NS).

### Effectiveness, Treatment Persistence, and Dose Escalation

**Figure 1.**
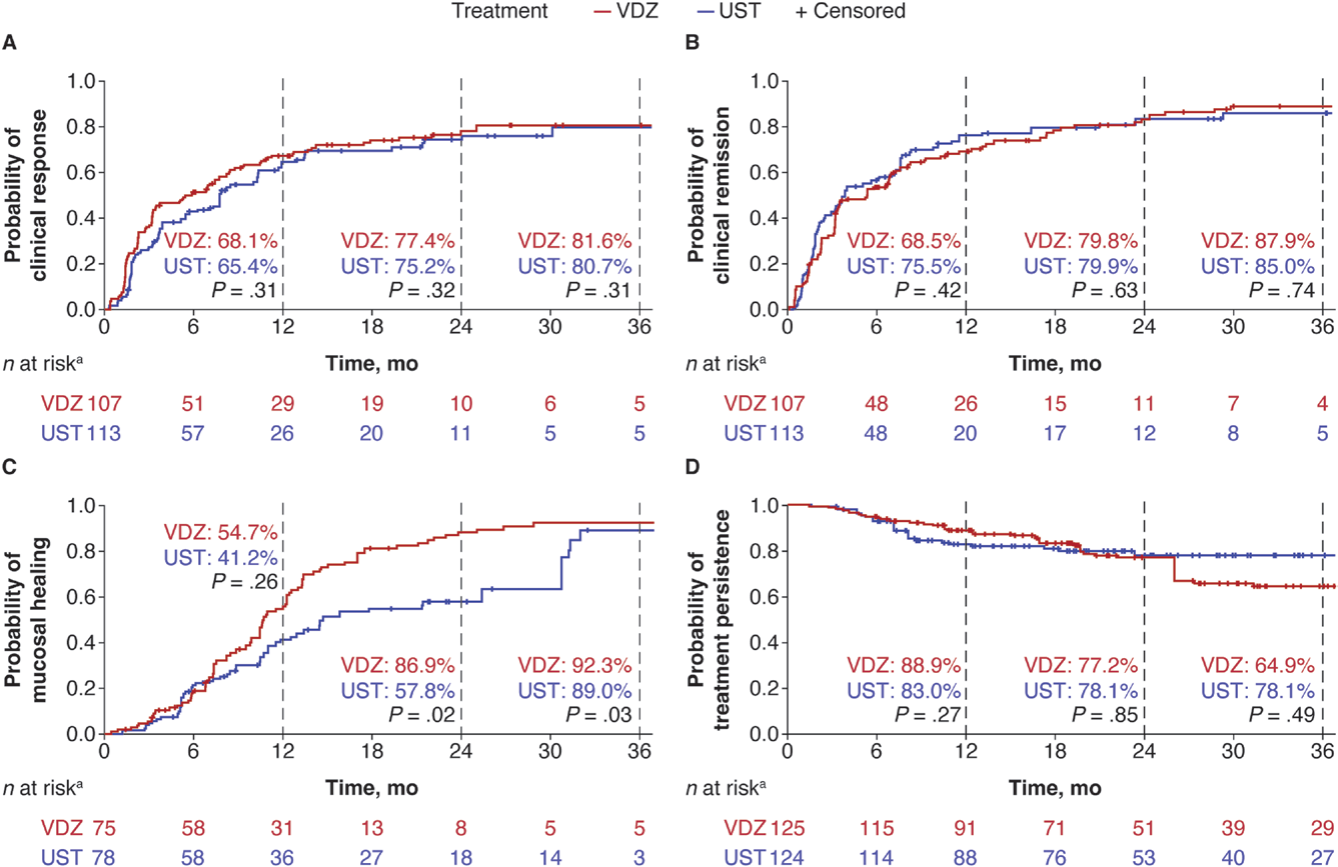
Weighted cumulative (A) clinical response, (B) clinical remission, (C) mucosal healing, and (D) treatment persistence over 36 months in patients with early (≤2 years) Crohn’s disease treated with vedolizumab (VDZ) and ustekinumab (UST). *P* values are adjusted using the inverse probability of treatment weighting model. ^a^*n* at risk is the sum of patient weights for each group of patients still receiving treatment who have clinical outcomes that can be assessed.

In patients with early CD, there were no significant differences over 36 months between vedolizumab and ustekinumab in the cumulative rates of clinical response (81.6% vs 80.7%; *P = *.31), clinical remission (87.9% vs 85.0%; *P* = .74), or treatment persistence (64.9% vs 78.1%; *P* = .49; Figure 1). In these patients, mucosal healing was significantly higher with vedolizumab than ustekinumab at both 24 (86.9% vs 57.8%; *P* = .02) and 36 months (92.3% vs 89.0%; *P* = .03; Figure 1C). There were no significant differences between vedolizumab and ustekinumab in the proportion of patients with dose escalation (19.5% vs 21.6%; *P* = .999).

**Figure 2.**
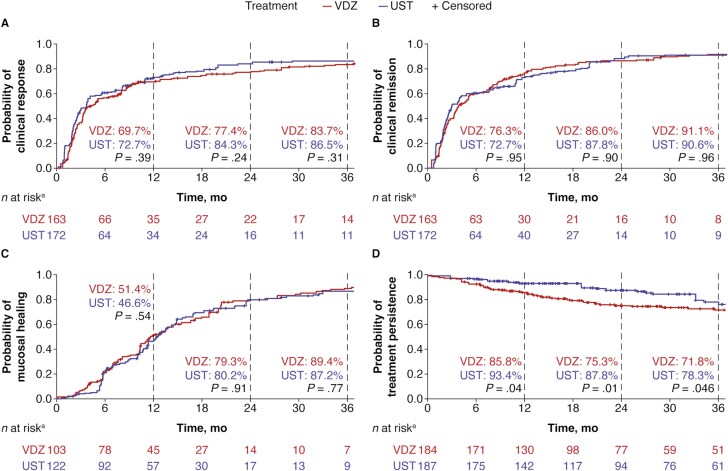
Weighted cumulative (A) clinical response, (B) clinical remission, (C) mucosal healing, and (D) treatment persistence over 36 months in patients with late (>2 years) Crohn’s disease treated with vedolizumab (VDZ) and ustekinumab (UST). *P* values are adjusted using the inverse probability of treatment weighting model. ^a^*n* at risk is the sum of patient weights for each group of patients still receiving treatment who have clinical outcomes that can be assessed.

In patients with late CD, there were no significant differences over 36 months between vedolizumab and ustekinumab in the cumulative rates of clinical response (83.7% vs 86.5%; *P* = .31), clinical remission (91.1% vs 90.6%; *P* = .96), or mucosal healing (89.4% vs 87.2%; *P* = .77; Figure 2). Treatment persistence was significantly higher with ustekinumab than with vedolizumab at 12, 24, and 36 months (Figure 2C). The proportion of patients with dose escalation was significantly different between vedolizumab and ustekinumab (25.3% vs 9.7%, respectively; *P* = .003).

### Safety and Healthcare Resource Use

**Figure 3.**
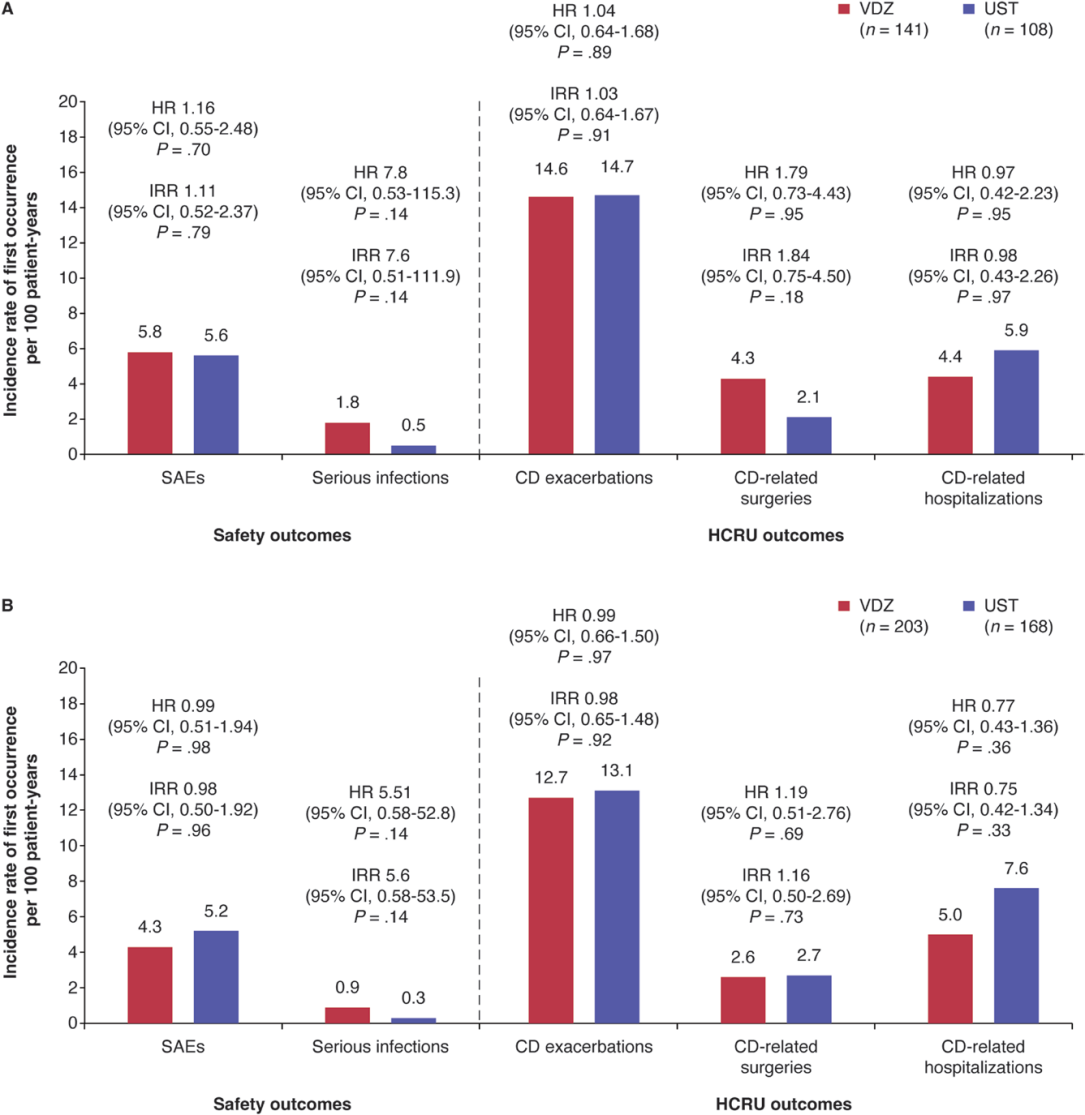
Serious adverse events (SAEs), serious infections, Crohn’s disease (CD) exacerbations, CD-related surgeries, and CD-related hospitalizations over 36 months in patients with (A) early (≤2 years) CD and (B) late (>2 years) CD treated with vedolizumab (VDZ) and ustekinumab (UST). HR, hazard ratio; IRR, incident rate ratio.

The risks of first SAEs (early CD, HR 1.16 [95% CI: 0.55-2.48]; late CD, HR 0.99 [95% CI: 0.51-1.94]) and first SIs (early CD, HR 7.8 [95% CI: 0.53-115.3]; late CD, HR 5.51 [95% CI: 0.58-52.8]) over 36 months were similar between vedolizumab and ustekinumab in both patients with early and late CD (Figure 3). HCRU outcomes were also similar between treatments in both cohorts (Figure 3).

## Discussion

This study demonstrates that vedolizumab and ustekinumab are highly effective in treating patients with both early and late CD. Across both subgroups of early and late CD, no statistically significant differences were observed between vedolizumab and ustekinumab with respect to clinical response, clinical remission rates, safety profiles, or HCRU over the 36 months. These findings suggest comparable effectiveness and safety profiles for both biologic agents in managing CD across different disease stages, offering valuable insights for clinicians in selecting appropriate treatment options for their patients.

There is accumulating evidence that for patients with moderate to severe CD, early use of biologic therapy can improve rates of clinical remission and mucosal healing as well as reduce disease complications. This is supported by a systematic review and meta-analysis that compared clinical remission and mucosal healing rates between early biologic treatment (<2 years of disease duration or top-down treatment strategy) and late/conventional treatment (biologic use after >2 years of disease duration or conventional step-up treatment strategy) in adult and pediatric patients.^19^ The analysis found that adults were approximately twice as likely to achieve clinical remission or mucosal healing at week 26 with early biologic therapy versus late/conventional treatment.^19^

In this study, there was a high rate of clinical remission (≥85%) in patients with early and late CD treated with vedolizumab or ustekinumab over 36 months. This compares well with the VEDOIBD registry study, which showed that in biologic-naïve CD patients, the rate of clinical remission was significantly higher in vedolizumab-treated patients after 2 years of maintenance therapy than those treated with an anti-TNF agent (64.2% vs 44.7%; *P* < .05).^20^

Mucosal healing is a long-term and objective treatment target in patients with CD and is thought to favorably change the natural course of CD.^21,22^ In the present study, patients with early CD who initiated treatment with vedolizumab had significantly higher rates of mucosal healing over 36 months than those treated with ustekinumab. Rates of mucosal healing were similar between treatments in patients with late CD. Our results further support the early use of vedolizumab in patients with CD to achieve mucosal healing. It remains to be determined if vedolizumab has a unique effect on mucosal healing, but it specifically inhibits the migration of α_4_β_7_-expressing memory T cells from the vascular space into the inflamed gut mucosa. This is thought to reduce mucosal inflammation by preventing the influx of new memory cells and local differentiation of effector cells.^23^

Treatment persistence is considered a proxy for effectiveness and safety in real-world studies,^24^ and is key to achieving long-term therapy outcomes. In our study, treatment persistence was numerically higher with ustekinumab than with vedolizumab in both the early (78.1% vs 64.9%) and late (78.3% vs 71.8%) CD cohorts but was only significantly different in the late CD cohort. Given the higher rate of vedolizumab-treated patients with early CD achieving mucosal healing, which is the long-term treatment goal for CD, it remains unclear why treatment persistence was higher in the ustekinumab group compared with the vedolizumab group. These results may reflect the low number of patients in the early CD cohort who had persistence outcomes at 36 months (vedolizumab, *n* = 29; ustekinumab, *n* = 27). In addition, the higher rates of treatment persistence with ustekinumab may be due to the route of administration of each agent in the EVOLVE Expansion study, with ustekinumab being administered subcutaneously during maintenance and vedolizumab being administered intravenously. Subcutaneous administration of vedolizumab was not available in the participating countries at the time of study initiation. Clinicians and patients have previously indicated a preference for subcutaneous over intravenous drug administration due to the convenience of self-administration at home.^25^ Additionally in our experience, many patients were switched to subcutaneous delivery of biological agents during the COVID-19 pandemic in an effort to reduce hospital visits, which may have affected treatment persistence with ustekinumab. Another consideration for treatment persistence was the dosing regimen for ustekinumab every 8 weeks, which is more frequent than the label recommendation in Belgium^26^ and Switzerland^27^ of every 12 weeks, although 8 weeks is permitted based on clinical judgment. Dosing every 8 weeks in this study is consistent with the label recommendations for vedolizumab in Australia, Belgium, and Switzerland.

In this study, the incidence rates per 100 person-years for first SAEs (vedolizumab, 4.3%-5.8%; ustekinumab, 5.2%-5.6%) and first SIs (vedolizumab, 0.9%-1.8%; ustekinumab, 0.3%-0.5%) were low for both treatments, which is in line with previous clinical trials that demonstrated generally well-tolerated profiles for long-term therapy with vedolizumab or ustekinumab.^17,28^ All HCRU outcomes were similar between treatments in both patient subgroups. In contrast to our findings that CD-related surgery rates were similar between treatments, in the retrospective observational SOJOURN study of CD (*n* = 1122), the cumulative incidence of CD-related surgery was 7.7% of vedolizumab-treated patients versus 11.6% of ustekinumab-treated patients. In addition, after adjusting for baseline characteristics, vedolizumab was associated with a significantly lower hazard rate of CD-related surgery than ustekinumab (adjusted HR 0.658 [95% CI: 0.436-0.994]; *P* = .047).^29^

This study had several limitations that should be noted. First, owing to the retrospective nature of the study there was no randomization, and only data from patient medical charts were available, with the data quality varying between sites. Second, there was no comparison with an anti-TNF agent. Third, as the effectiveness algorithms for clinical response, clinical remission, and mucosal healing were used according to data availability in medical charts, assessments for some patients were not as stringent as those in controlled clinical trials (eg, endoscopic data were not available for approximately 39% of patients in the early and late subgroups). Fourth, there were some differences in the unadjusted baseline characteristics between the vedolizumab and ustekinumab cohorts, which were addressed by using propensity score–based IPTW methodology to compare outcomes in weighted patients treated with vedolizumab and ustekinumab. However, it was possible that there were some unmeasured confounders, which may have led to residual confounding. Fifth, no statistical comparisons were made between the early and late CD subgroups; therefore, any differences between the subgroups are hypothesis testing.^30^ Finally, the study findings may not be generalizable to certain geographies due to differences in treatment availabilities and prescribing patterns, but this is partially offset by the inclusion of data from 3 different countries and various types of sites included in the EVOLVE Expansion study.

## Conclusions

In this study, distinct treatment outcomes were observed between vedolizumab and ustekinumab in patients with early- and late-stage CD. In patients with early CD, vedolizumab demonstrated significantly higher rates of mucosal healing compared with ustekinumab over the 36-month follow-up period. Conversely, in patients with late CD, treatment persistence was significantly higher in those receiving ustekinumab compared with vedolizumab. These results suggest that vedolizumab might be preferable in patients with early CD where mucosal healing is the therapeutic objective. However, treatment choice will ultimately depend on individual patient characteristics, patient and physician preferences, and specific clinical goals. Despite the differences in mucosal healing and treatment persistence, our analysis revealed no significant differences between vedolizumab and ustekinumab in clinical response, clinical remission, safety profile, or HCRU over the 36-month duration, regardless of disease stage. These findings indicate that both vedolizumab and ustekinumab could be considered viable treatment options for both early- and late-stage CD, providing clinicians with valuable insights into personalized therapeutic strategies for this complex condition.

## Supplementary Material

otaf031_suppl_Supplementary_Figure_S1

## Data Availability

The datasets, including the redacted study protocol, redacted statistical analysis plan, and individual participants data supporting the results reported in this article, will be made available within 3 months from the initial request to researchers who provide a methodologically sound proposal. The data will be provided after its deidentification, in compliance with applicable privacy laws, data protection, and requirements for consent and anonymization.

## Clinical Trial Registration

The EVOLVE Expansion study was registered at ClinicalTrials.gov; NCT05056441.
