# Supplementary material for: Real-World Clinical Effectiveness and Safety of Vedolizumab and Ustekinumab in Biologic-Naïve Patients With Early or Late Crohn’s Disease: Results From the EVOLVE Expansion Study
Source: Crohns Colitis 360. 2025 Jul 9;7(3):otaf031. doi: 10.1093/crocol/otaf031 (PMC12238940; doi:10.1093/crocol/otaf031)

**Real-World Clinical Effectiveness and Safety of Vedolizumab and Ustekinumab in Biologic-Naïve Patients With Early or Late Crohn’s Disease: Results From the EVOLVE Expansion Study**

**Supplementary Data**

Supplementary Figure S1. Patient flow diagram. ^a^*n* reflects the number of patients who satisfied a specific criterion and may not be exclusive between criteria. ^b^Initiation of treatment with vedolizumab or ustekinumab. CD, Crohn’s disease.


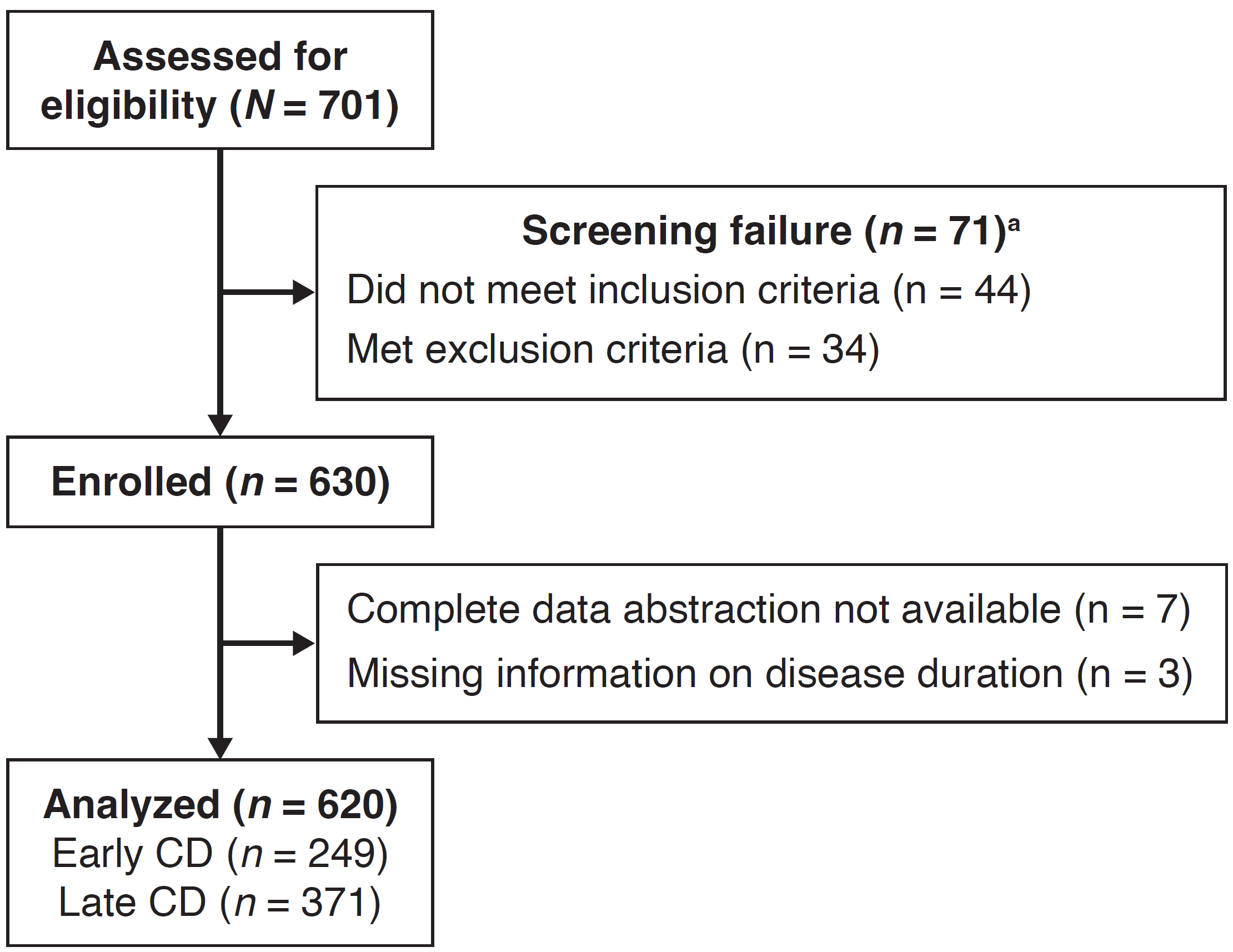

Supplement: otaf031_suppl_Supplementary_Figure_S1 [file otaf031_suppl_supplementary_figure_s1.docx]
